# The Therapeutic Potential of Nanoparticles to Reduce Inflammation in Atherosclerosis

**DOI:** 10.3390/biom9090416

**Published:** 2019-08-26

**Authors:** Armita Mahdavi Gorabi, Nasim Kiaie, Željko Reiner, Federico Carbone, Fabrizio Montecucco, Amirhossein Sahebkar

**Affiliations:** 1 Research Center for Advanced Technologies in Cardiovascular Medicine, Tehran Heart Center, Tehran University of Medical Sciences, 1411713138 Tehran, Iran; 2 University Hospital Centre Zagreb, School of Medicine University of Zagreb, Department of Internal Medicine, 1000 Zagreb, Croatia; 3 First Clinic of Internal Medicine, Department of Internal Medicine, University of Genoa, 16132 Genoa, Italy; 4 IRCCS Ospedale Policlinico San Martino Genoa - Italian Cardiovascular Network, 16132 Genoa, Italy; 5 First Clinic of Internal Medicine, Department of Internal Medicine, University of Genoa, 16132 Genoa, Italy and Centre of Excellence for Biomedical Research (CEBR), University of Genoa, 16132 Genoa, Italy; 6 Neurogenic Inflammation Research Center, Mashhad University of Medical Sciences, Mashhad, Iran; 7 Biotechnology Research Center, Pharmaceutical Technology Institute, Mashhad University of Medical Sciences, Mashhad, Iran; 8 School of Pharmacy, Mashhad University of Medical Sciences, 9177948564 Mashhad, Iran

**Keywords:** atherosclerosis, cardiovascular disease, nanoparticles, drug delivery, inflammation, immune system

## Abstract

Chronic inflammation is one of the main determinants of atherogenesis. The traditional medications for treatment of atherosclerosis are not very efficient in targeting atherosclerotic inflammation. Most of these drugs are non-selective, anti-inflammatory and immunosuppressive agents that have adverse effects and very limited anti-atherosclerotic effects, which limits their systemic administration. New approaches using nanoparticles have been investigated to specifically deliver therapeutic agents directly on atherosclerotic lesions. The use of drug delivery systems, such as polymeric nanoparticles, liposomes, and carbon nanotubes are attractive strategies, but some limitations exist. For instance, nanoparticles may alter the drug kinetics, based on the pathophysiological mechanisms of the diseases. In this review, we will update pathophysiological evidence for the use of nanoparticles to reduce inflammation and potentially prevent atherogenesis in different experimental models.

## 1. Introduction

Atherosclerosis is a largely-investigated multifactorial disease. The pathophysiology of the disease has partially been attributed to changed immune system functions. Immune cells, including lymphocytes, macrophages and neutrophils, found in atherosclerotic lesions suggest an important role of inflammation in the development of atherosclerosis [1,2]. Furthermore, inflammation due to autoimmune processes and infectious diseases may precipitate the atherogenesis. The development of carriers precisely delivering the therapeutic compounds to the target sites is a major goal in the modern medicine. This approach may minimize the potential adverse effects and be more effective in treating the lesions. Among different approaches, drug delivery systems by nanoparticles may be very promising [3]. Nanoparticles have been used for actively targeting the atherosclerotic lesions [4]. In this review, we will update evidence on the role of nanoparticles for directly reducing inflammation in atherogenesis.

## 2. Treatments Targeting Inflammation in Atherogenesis

According to the “classical” pathophysiological view, atherosclerosis occurs because of lipid accumulation in the vessel wall. Nonetheless, according to the current view, atherosclerosis is a low-grade chronic inflammatory disease, in which the immune system plays a central role in the initiation, progress and stability of lesions [5,6,7]. The clinical manifestations are due to rupture/erosion of atherosclerotic plaques, which is followed by thrombosis, and eventually vessel lumen obstruction [8]. Inflammatory degrading enzymes, such as matrix metalloproteinases (MMPs), which are released by immune cells, can favor plaque fissuring, erosion and instability [9,10]. Those mediators can be targeted by selective anti-inflammatory treatments both in primary and secondary prevention of CV diseases. The currently available therapeutic options for athero-prevention are directed towards reduction of classical risk factor, such as smoking, hypertension, and dyslipidemia. Statins were shown to inhibit the endogenous synthesis of cholesterol, primarily in the hepatic cells, but have also pleiotropic effects [11,12,13]. For instance, these drugs can enhance endothelial dysfunction, adhesion of leukocytes to the endothelium, infiltration of LDL particles into the sub-endothelial space [14,15,16].

Therefore, statins can act against cholesterol and non-specific atherosclerotic inflammation at the same time. Recently, some studies have indicated that the IL-1 signaling pathway can be a potential target of more selective anti-inflammatory drugs. In a clinical trial, treatment inhibiting this pathway with a monoclonal antibody against IL-1β (canakinumab) was investigated with promising results [17,18]. Another anti-inflammatory drug, methotrexate (MTX), which is used as anti-inflammatory drug in autoimmune inflammatory disorders, was suggested to decrease the risk of cardiovascular diseases in subjects who were in a prolonged inflammatory state [19,20]. MTX has also been shown to decrease macrophage recruitment to the vessel wall and to have beneficially effects on atherogenesis in experimental animals [21]. In spite of uncertainties concerning the mechanisms by which MTX might have effects on atherogenesis and its adverse effects, it has been reported that it can downregulate the synthesis of pro-inflammatory mediators and adhesion molecules and has effects on both endothelial and immune cells [14].

Although anti-inflammatory drugs have been associated with beneficial effects, systemic use of such drugs is limited because of their adverse effects, such as neutropenia, bone marrow suppression, and immunosuppression. Stimulation or inhibition of the inflammatory process may be beneficial but also harmful depending upon the phase of the atherosclerosis development [22]. It seems that further efforts are needed to come up with approaches, which would be beneficial but would modulate the immune system to minimize side effects. Of course, the ideal drug should be able to be effective in the majority of the different phases of atherogenesis. It was reported that resolvin E1 help in the resolution of inflammation, with beneficial effects on atherosclerotic plaques in both early and advanced stage of atherosclerotic disease [23]. However, another strategy can be controlling of the secretion or activation of agents utilized that might be involved in atherosclerotic lesions development and/or cardiovascular outcomes. Such strategies may consist of using glutamyl-modified compounds for controlling the high levels of gamma-glutamyl transferase (gGT) enzyme in atherosclerotic plaques [24], controlling the high cholesterol content of the lesions or modulation of the pathways involved in the transformation of monocytes/macrophages into foam cells in the vessel walls [25].

## 3. The Potential of Nanoparticles as to Prevent and Treat Atherosclerosis and Related Complications

In order to reduce adverse effects and improve comfortability of administration, researchers designed well-organized and targeted delivery approaches for anti-inflammatory agents, such as those that can be encapsulated into nanoparticles [26]. Various types of nanoparticles have been developed for drug delivery (Table 1). The properties and production techniques for a number of these compounds have already been characterized [27,28,29,30]. However, there are also some disadvantages and limitations for the clinical use. Nanoparticles are based upon limited possibilities to fully control their metabolism, since they are usually accumulated in the reticuloendothelial system (RES). In addition, they are known for the limited reproducibility, high price of production, particularly when multifunctional abilities are desired [27,31]. Nevertheless, characteristics of the nanoparticles can be controlled during their production that permits the optimization of the conjugated drug and the specificity of the target for nanoparticles [30,32]. When atherosclerosis is clinically manifested, nanoparticles conjugated with anti-inflammatory compounds could be an efficient approach to target pro-inflammatory mediators within atherosclerotic plaques, potentially minimizing adverse effects of the drugs [33,34]. Nanoparticles conjugated or loaded with anti-inflammatory drugs can modulate inflammatory and vascular cell functions. Simultaneously, nanoparticles can also be conjugated with substances used for plaque imaging, facilitating the identification of unstable atherosclerotic plaques. These multifunctional agents referred to as theranostics can target macrophage, integrin ανβ3 and VCAM-1 to deliver imaging and therapeutic agent specific sites of plaques [35]. Dextran-coated iron oxide nanoparticles, gold nanorods, carbon nanotubes, hyaluronic acid-polypyrrole nanoparticles, hybrid lipid-latex nanoparticles and liposomes are some examples of nanoparticles used for targeting macrophages and simultaneous delivery of drugs. Similarly, paramagnetic nanoparticles targeting integrin ανβ3 and magnetic microbubbles targeting VCAM-1 have been reported in the literature. Incorporation of antibodies such as CD11b and anti-VCAM1, or molecules such as dextran and mannose in theranostic agents for the targeting of macrophages in the plaque has also been reported [36]. Some similar approaches, using imaging techniques for featuring components of atherogenesis, such as magnetic resonance imaging (MRI), optical imaging (OI), ultrasound and photoacoustic (US-PA), nuclear imaging based on single photon and positron emission tomography (SPECT, PET), and computed tomography (CT) are already under investigation [19,36,37,38,39,40]. Although the specificity of nanoparticle targeting has not been fully described, a number of suggestions has been developed. The changed hemodynamic forces at the sites of plaques development may be susceptible for the deposition of nanoparticles [41]. There are spaces between the endothelial cells that permit penetration of small particles [37]. In addition, immune cells may be involved in delivering nanoparticles to the sites of inflammation [42].

After the administration of nanoparticles in vivo, a protein corona coats the nanoparticles, causing changes in their biological characteristics. Development of coating by protein corona is usually considered as the first phase in the nanoparticle sequestration by the RES. Different strategies have been developed to inhibit this event. Currently available approaches are based upon controlling the stiffness of nanoparticles, since deformable particles are less susceptible of being internalized by macrophages in RES in off-target sites [43,44]. Another approach is based upon functionalization and coatings of the protein corona by substances such as polyethylene glycol (PEG). It seems that PEG is an antifouling substance, which can affect the composition of the protein corona [45]. In addition, PEG can stimulate immune response, but it needs the addition of some other functionalizing compounds such as ganglioside which can attenuate the immunogenicity of PEGylated liposomes while preserving their therapeutic efficacy [46]. Peptides can also be considered as potential coating compounds (zwitterionic peptides) to minimize the serum-protein adsorption effect [47]. Another example are aptamer-like peptides, used to enrich the protein corona with specific compounds in the biological fluids. When favorably organized, these molecules might be promising [48]. Such methods might ensure an increased half-life for nanoparticles in the biological fluids.

Nanoparticles can be also activated via radiofrequency or photothermal energy. For instance, a potential application for inhibiting macrophages was represented by laser pulse excitation of iron oxide nanoparticles layered with dextran and gold within cells. Nanoparticles were investigated also for potential photothermolysis as well as MRI [49]. The same technique was evaluated in a clinical trial, in which nanoparticles were constructed as silica shells coated with gold. Magnetic nanoparticles were administered into atherosclerotic plaques using on-artery patch or magnetic orienting technique. Nanoparticle deterioration through a near-infrared (NIR) laser resulted in a remarkable decrease in the size of atherosclerotic plaques and the event free survival of the clinical follow-up showed a significantly lower risk of cardiovascular death in the group which received nanoparticles when compared to controls [50].

The gene regulation approach has also been hypothesized to be useful in the prevention and therapy of atherosclerosis when using nanoparticles. In a study, small interfering RNA (siRNA)-loaded nanoparticles were used to downregulate the expression of C–C chemokine receptor type 2 (CCR-2), which is involved in the infiltration of inflammatory monocytes to the sites of both atherosclerotic plaque and ischemic myocardial injury [51,52]. Photon and positron emission (PET) signaling was decreased from 89Zr-labeled dextran nanoparticles when compared with the mice administered with other siRNAs [53]. Furthermore, when microRNA-712 (targeting VCAM-1) was delivered by cationic lipid nanoparticles to ApoE^−/−^ mice, there was a downregulation of the tissue inhibitor of metalloproteinase 3 (TIMP3) gene. The administration of the nanoparticles resulted in slowing down of the plaque progression [54]. Similarly, the silencing of Src homology region 2 domain-containing tyrosine phosphatase-1 (SHP-1) in cardiomyocytes suppress their apoptosis under hypoxia [55].

Another approach could be inhibiting the infiltration of monocytes to the site of the atherosclerotic plaques as well as blocking their transformation into macrophages. Nanoparticles conjugated with pioglitazone, which is an agonist of the peroxisome proliferator-activated receptor (PPARγ) and is involved in the regulation of the fatty acids, could modulate the differentiation of macrophages. Such nanoparticles were evaluated in the ApoE^−/−^ mice on a high fat diet (HFD) and treated with angiotensin II, leading to a monocyte-mediated inflammation. Two days after administration of nanoparticles, the balance of non- and pro-inflammatory monocytes was favorably changed—mainly towards non-inflammatory macrophages. Atherosclerotic plaques were more stable and the rupture risk was decreased [29,56]. The ability of MTX-loaded nanoparticles to prevent the development of an inflammatory state was also reported [40]. Nanoparticles were selectively absorbed by macrophages in the lipid-rich plaques. On the other side, in the ApoE^−/−^ mice fed with HFD diet and to whom MTX-conjugated nanoparticles were administered, there were much less atherosclerotic lesions in the aortic arch and even more effective was the treatment when MTX was combined with etoposide [20,57]. The combination of MTX with different other bioactive agents has been analyzed as well. The combination of MTX-conjugated nanoparticles and paclitaxel-conjugated LDL-mimicking nanoparticles was investigated [20]. There was a reduction in the atherosclerotic lesion size and improving of the intima thickness in the New Zealand white rabbits on atherogenic diet when they were administered with MTX-conjugated nanoparticles and paclitaxel-conjugated LDL-mimicking nanoparticles. Similar cytotoxic effects were also reported for other compounds used in chemotherapy therapies such as glucocorticoid and doxorubicin [58,59,60]. It seems that the beneficial effects of this combination were achieved because of decreased function of the macrophages and that the use of combined chemotherapy in nanoparticles can achieve stronger effects on highly inflamed atherosclerotic lesions.

**Figure 1:**
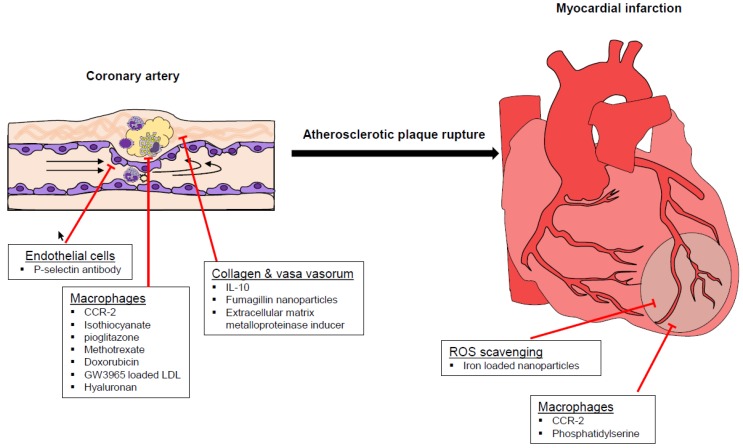
Studies on nanoparticles in atherosclerosis cover different steps of atherosclerotic disease from the early development of fatty streaks to the athero-thrombotic consequences. Current approaches mainly involve endothelial cells, extracellular matrix and especially macrophage recruitment and function. CCR: C-C chemokine receptor; IL-interleukin; ROS: Reactive oxygen species.

Nonetheless, such drugs are burdened by a non-specific systemic decrease of macrophage with consequent risk of infections. Non-ablation approaches targeting macrophage functionality have then developed to reduce side effects. Magnetic microbubbles conjugated with P-selectin are effective in the early phase of atherosclerosis, when the shear stress generates fatty streaks [61]. Similarly, targeting angiogenesis may prevent systemic complications. Integrin-targeted paramagnetic nanoparticles loading with fumagillin are effective in stabilizing or even regressing atherosclerotic plaque trough suppressing vasa vasorum expansion [62,63]. Another approach to prevent atherosclerotic complications and progression of ischemic myocardial injury relies on reactive oxidative species scavenging nanoparticles [64]. The use of phosphatidylserine (PS)-presenting liposome was shown to provide additional benefit in post-myocardial infarction injury by down-regulating macrophage activation. Being typically expressed on apoptotic cells, PS triggers a “non-inflammatory” clearance by macrophage with prevalent secretion of tumor growth factor-β and interleukin-10 instead of tumor necrosis factor-α [65]. (Table 1) (Figure 1).

**Table 1:** Recent findings on the application of nanoparticles in the therapy of atherosclerosis and related complications.

Nanoparticle	Target	Outcome	Ref
**siRNA**			
siRNA targeting CCR2	Monocytes, macrophages.	Reduction of atherosclerosis Attenuated infarct inflammation, post-infarction left ventricular remodeling	[51,52]
**Sulphate-based nanoparticles**			
Nanoparticles loaded with fluorescein isothiocyanate and/or pioglitazone.	Monocytes, macrophages.	Modified polarity of monocytes in the periphery. Decreased development of inflammatory macrophages. Destabilized atherosclerotic plaque and rupture.	[56]
**Lipid-based nanoparticles**			
Lipid coated nanoparticles loaded with MTX	Macrophages, foam cells	Decreased plaque coverage in the aortic arch	[40]
Library of LDL mimicking nanoparticles loaded with GW3965	Monocytes and Macrophages for reversing cholesterol efflux.	Decreased total lipids in aortic macrophages. Decreased monocyte number.	[66]
Lipid core nanoparticles carrying MTX and/or PTX	Macrophages	Decreased size of the plaque and of intima area. Reduced number of macrophages in aortic lesions. Downregulation of MMP-9 and TNF-α.	[20]
Liposomal nanoparticles loaded with prednisolone	Macrophage lipid loading, ER stress and apoptosis	Lipotoxicity	[59]
Lipid core nanoparticles carrying doxorubicin	Macrophages	Anti-inflammatory and anti-proliferating effects	[60]
Liposomes presenting PS	Macrophages	Shift toward anti-inflammatory phenotype with consequent improvement of myocardial healing	[65]
**Glycosaminoglycan**			
Hyaluronan nanoparticles	Atherosclerotic plaque, macrophages	Decreased size of the atherosclerotic lesions. Decreased macrophage number. Increased collagen content.	[58,67]
**Other approaches**			
Nanoparticles loaded with the EMMPRIN (extracellular matrix metalloproteinase inducer) Ldlr, low density lipoprotein receptor) binding peptide AP-9.	EMMPRIN	Ameliorated heart contractility. Decreased cardiac necrosis. Decreased levels of MMP-2 and MMP-9	[68]
Nanoparticles containing IL-10 and targeting peptide collagen IV	Collagen IV	Reduced oxidative stress in lesions. Stabilized atherosclerotic plaques.	[69]
Magnetic microbubbles modified with P-selectin antibody	Endothelial cells	Leukocyte rolling	[61]
Fumagillin nanoparticles	Vasa vasorum	Reduced neovascularization	[62,63]
Iron oxide–cerium oxide core–shell nanoparticles	Macrophages	ROS scavenging with reduced atherosclerotic burden and improved myocardial healing	[64]

MTX, methotrexate; LDL, low-density lipoprotein; PTX, Paclitaxel; MMP, matrix metalloproteinase; TNF, tumor necrosis factor; IL-10, interleukin-10; ER, endothelial reticulum; ROS: Reactive oxygen species; PS: Phosphatidylserine.

## 4. Future Perspectives in the Application of Nanoparticles in the Prevention and Treatment of Atherosclerosis

The use of nanoparticles to deliver anti-inflammatory substances has been recently investigated, suggesting promising results. A number of clinical trials is evaluating the application of nanoparticles and anti-inflammatory substances to reduce the number and size of atherosclerotic plaques or to stabilize them. Nevertheless, further basic research seems to be necessary to learn more about the underlying functional mechanisms of the nanoparticles. This is necessary to encourage more well-designed clinical trials, which would use anti-inflammatory drugs and nanoparticles to prevent or treat atherosclerosis. Novel strategies that look promising are those on mechanisms of interaction between immune cells and nanoparticles, especially with respect to the fate of nanoparticles as well as their components after deposition at site of atherosclerotic lesions and the effects on immune cells. Such studies will hopefully help constructing nanoparticles tailored for specific accumulation and controlled activation of the delivered substance. On the other side, the exact quality and quantity of nanoparticles absorption by immune cells has not been fully understood. There are various absorption pathways in similar phenotypes of immune cells [70], and the exocytosis of immune cells which is significant in the removal of nanoparticles with drugs and contrast substances from the body in still unclear [71]. In the meantime, a number of animal models have been designed, which simulate various phases of atherosclerosis development [72,73].

## 5. Conclusions

Atherosclerosis is a disease which cannot be easily detected with the available imaging techniques in the initial steps of development. Currently available therapeutic strategies are aimed at the systemic, but not local and targeted, prevention and treatment of atherosclerosis. All of them have limited efficacy and some adverse effects. Targeted delivery of diagnostic contrast substances or therapeutic drugs by nanoparticles to sites of incipient atherosclerotic lesions is considered to be a sophisticated strategy for the diagnosis as well as prevention and therapy of atherosclerosis. Targeted delivering approach using nanoparticles can promote the stability and bioavailability of the drugs, improve the detection sensitivity, enhance the therapeutic efficacy, improve the pharmacokinetics of the drugs, and reduce the adverse systemic side effects. Nevertheless, there are still a number of drawbacks in such nanoparticles with respect to stability, structure design, toxicity, targeting efficacy, and production, requiring optimization to devise nanoparticle-based therapeutic/diagnostic approaches for atherosclerosis that are clinically favorable. Application of porous compounds combined with imaging agents is an interesting option and studies in the future need to concentrate on enhancing its stability and efficacy in vitro and in-vivo. It has to be emphasized that possible beneficial effects of nanoparticles have largely been obtained from the in vitro experiments and experiments using animal models. Therefore, it will be a challenging effort to translate the results of these studies into possible clinical use. Current nanoparticle-based strategies have mainly focused on the anti-inflammatory effects by targeting the development of macrophages/foam cells as well as the recruitment of monocytes to the atherosclerotic plaques. However, gene-therapy, monoclonal antibodies, and combination therapies can be promising if merged with the potential beneficial use of nanoparticles. Moreover, multifunctional nanoparticles can be developed that could facilitate the imaging and targeted delivery of drugs in humans, i.e., in a clinical setting. Although the application of nanoparticle technology in the prevention and treatment of atherosclerosis is an emerging field, the progresses and the results obtained so far have been promising. This might open new horizons in the therapy of atherosclerotic cardiovascular diseases.

## Abbreviations

ApoE^−^/^−^	apolipoprotein E(Apoe) knockout
CCR	C-C chemokine receptor
CT	computed tomography
CV	cardiovascular
ER	endothelial reticulum
gGT	gamma-glutamyl transferase
HFD	high fat diet
IL-10	interleukin-10
LDL	low-density lipoprotein
Ldlr	low density lipoprotein receptor
MMP	matrix metalloproteinase
MMP	matrix metalloproteinase
MRI	magnetic resonance imaging
MTX	methotrexate
NIR	near-infrared
OI	optical imaging
PEG	polyethylene glycol
PET	positron emission tomography
PPARγ	peroxisome proliferator-activated receptor
PS	phosphatidylserine
PTX	Paclitaxel
RES	reticuloendothelial system
ROS	Reactive oxygen species
SHP-1	Src homology region 2 domain-containing tyrosine phosphatase-1
siRNA	small interfering RNA
SPECT	single photon emission tomography
TIMP3	tissue inhibitor of metalloproteinase 3
TNF	tumor necrosis factor
US-PA	ultrasound and photoacoustic
VCAM-1	vascular cell adhesion molecule 1
Zr	Zirconium

## References

[1] Shoenfeld Y., Sherer Y., Harats D. (2001). Atherosclerosis as an infectious, inflammatory and autoimmune disease. Trends Immunol..

[2] Ross R. (1999). Atherosclerosis—An inflammatory disease. New Engl. J. Med..

[3] De Jong W.H., Borm P.J. (2008). Drug delivery and nanoparticles: Applications and hazards. Int J. Nanomed..

[4] Bejarano J., Navarro-Marquez M., Morales-Zavala F., Morales J.O., Garcia-Carvajal I., Araya-Fuentes E., Flores Y., Verdejo H.E., Castro P.F., Lavandero S. (2018). Nanoparticles for diagnosis and therapy of atherosclerosis and myocardial infarction: Evolution toward prospective theranostic approaches. Theranostics.

[5] Libby P. (2012). Inflammation in atherosclerosis. Arterioscler. Thromb. Vasc. Biol..

[6] Hoseini Z., Sepahvand F., Rashidi B., Sahebkar A., Masoudifar A., Mirzaei H. (2018). NLRP3 inflammasome: Its regulation and involvement in atherosclerosis. J. Cell Physiol..

[7] Parsamanesh N., Moossavi M., Bahrami A., Fereidouni M., Barreto G., Sahebkar A. (2019). NLRP3 inflammasome as a treatment target in atherosclerosis: A focus on statin therapy. Int. Immunopharmacol..

[8] Rothwell P., Gutnikov S., Warlow C. (2003). Reanalysis of the final results of the European Carotid Surgery Trial. Stroke.

[9] Everett B.M., Pradhan A.D., Solomon D.H., Paynter N., MacFadyen J., Zaharris E., Gupta M., Clearfield M., Libby P., Hasan A.A. (2013). Rationale and design of the Cardiovascular Inflammation Reduction Trial: A test of the inflammatory hypothesis of atherothrombosis. Am. Heart J..

[10] Finn A.V., Nakano M., Narula J., Kolodgie F.D., Virmani R. (2010). Concept of Vulnerable/Unstable Plaque. Arterioscler. Thromb Vasc. Biol..

[11] Parizadeh S.M.R., Azarpazhooh M.R., Moohebati M., Nematy M., Ghayour-Mobarhan M., Tavallaie S., Rahsepar A.A., Amini M., Sahebkar A., Mohammadi M. (2011). Simvastatin therapy reduces prooxidant-antioxidant balance: Results of a placebo-controlled cross-over trial. Lipids.

[12] Sahebkar A., Kotani K., Serban C., Ursoniu S., Mikhailidis D.P., Jones S.R., Ray K.K., Blaha M.J., Rysz J., Toth P.P. (2015). Statin therapy reduces plasma endothelin-1 concentrations: A meta-analysis of 15 randomized controlled trials. Atherosclerosis.

[13] Sahebkar A., Serban C., Mikhailidis D.P., Undas A., Lip G.Y.H., Muntner P., Bittner V., Ray K.K., Watts G.F., Hovingh G.K. (2015). Association between statin use and plasma d-dimer levels: A systematic review and meta-analysis of randomised controlled trials. Thromb. Haemost..

[14] Coomes E., Chan E.S., Reiss A.B. (2011). Methotrexate in atherogenesis and cholesterol metabolism. Cholesterol.

[15] Duivenvoorden R., Tang J., Cormode D.P., Mieszawska A.J., Izquierdo-Garcia D., Ozcan C., Otten M.J., Zaidi N., Lobatto M.E., van Rijs S.M. (2014). A statin-loaded reconstituted high-density lipoprotein nanoparticle inhibits atherosclerotic plaque inflammation. Nat. Commun..

[16] Tsujita K., Sugiyama S., Sumida H., Shimomura H., Yamashita T., Yamanaga K., Komura N., Sakamoto K., Oka H., Nakao K. (2015). Impact of Dual Lipid-Lowering Strategy With Ezetimibe and Atorvastatin on Coronary Plaque Regression in Patients With Percutaneous Coronary Intervention: The Multicenter Randomized Controlled PRECISE-IVUS Trial. J. Am. Coll. Cardiol..

[17] Ridker P.M., Everett B.M., Thuren T., MacFadyen J.G., Chang W.H., Ballantyne C., Fonseca F., Nicolau J., Koenig W., Anker S.D. (2017). Antiinflammatory Therapy with Canakinumab for Atherosclerotic Disease. N. Engl. J. Med..

[18] Ridker P.M., Thuren T., Zalewski A., Libby P.J.A.h.j. (2011). Interleukin-1β inhibition and the prevention of recurrent cardiovascular events: Rationale and design of the Canakinumab Anti-inflammatory Thrombosis Outcomes Study (CANTOS). Am. Heart J..

[19] Cervadoro A., Palomba R., Vergaro G., Cecchi R., Menichetti L., Decuzzi P., Emdin M., Luin S. (2018). Targeting Inflammation With Nanosized Drug Delivery Platforms in Cardiovascular Diseases: Immune Cell Modulation in Atherosclerosis. Front. Bioeng. Biotechnol..

[20] Gomes F.L.T., Maranhao R.C., Tavares E.R., Carvalho P.O., Higuchi M.L., Mattos F.R., Pitta F.G., Hatab S.A., Kalil-Filho R., Serrano C.V. (2018). Regression of Atherosclerotic Plaques of Cholesterol-Fed Rabbits by Combined Chemotherapy With Paclitaxel and Methotrexate Carried in Lipid Core Nanoparticles. J. Cardiovasc. Pharmacol. Ther..

[21] Bulgarelli A., Dias A.A.M., Caramelli B., Maranhao R.C. (2012). Treatment With Methotrexate Inhibits Atherogenesis in Cholesterol-Fed Rabbits. J. Cardiovasc. Pharm..

[22] Narasimhulu C.A., Fernandez-Ruiz I., Selvarajan K., Jiang X., Sengupta B., Riad A., Parthasarathy S.J.C.o.i.p. (2016). Atherosclerosis—Do we know enough already to prevent it?. Curr. Opin. Pharmacol..

[23] Libby P., Tabas I., Fredman G., Fisher E.A. (2014). Inflammation and its Resolution as Determinants of Acute Coronary Syndromes. Circ. Res..

[24] Belcastro E., Franzini M., Cianchetti S., Lorenzini E., Masotti S., Fierabracci V., Pucci A., Pompella A., Corti A. (2015). Monocytes/macrophages activation contributes to b-gamma-glutamyltransferase accumulation inside atherosclerotic plaques. J. Transl. Med..

[25] Rousselle A., Qadri F., Leukel L., Yilmaz R., Fontaine J.F., Sihn G., Bader M., Ahluwalia A., Duchene J. (2013). CXCL5 limits macrophage foam cell formation in atherosclerosis. J. Clin. Invest..

[26] Lima S.A.C., Reis S. (2015). Temperature-responsive polymeric nanospheres containing methotrexate and gold nanoparticles: A multi-drug system for theranostic in rheumatoid arthritis. Coll. Surf. B.

[27] Ulbrich K., Hola K., Subr V., Bakandritsos A., Tucek J., Zboril R. (2016). Targeted Drug Delivery with Polymers and Magnetic Nanoparticles: Covalent and Noncovalent Approaches, Release Control, and Clinical Studies. Chem Rev..

[28] Cheraghi M., Negahdari B., Daraee H., Eatemadi A. (2017). Heart targeted nanoliposomal/nanoparticles drug delivery: An updated review. Biomed. Pharmacother..

[29] Matoba T., Koga J., Nakano K., Egashira K., Tsutsui H. (2017). Nanoparticle-mediated drug delivery system for atherosclerotic cardiovascular disease. J. Cardiol..

[30] Allen S., Liu Y.G., Scott E. (2016). Engineering Nanomaterials to Address Cell-Mediated Inflammation in Atherosclerosis. Regen Eng. Transl. Med..

[31] Cheng Z., Al Zaki A., Hui J.Z., Muzykantov V.R., Tsourkas A. (2012). Multifunctional nanoparticles: Cost versus benefit of adding targeting and imaging capabilities. Science.

[32] Pentecost A.E., Lurier E.B., Spiller K.L. (2016). Nanoparticulate Systems for Controlling Monocyte/Macrophage Behavior. Microscale Technologies for Cell Engineering.

[33] Jokerst J.V., Gambhir S.S. (2011). Molecular imaging with theranostic nanoparticles. Acc. Chem. Res..

[34] Di Mascolo D., Lyon C.J., Aryal S., Ramirez M.R., Wang J., Candeloro P., Guindani M., Hsueh W.A., Decuzzi P. (2013). Rosiglitazone-loaded nanospheres for modulating macrophage-specific inflammation in obesity. J. Control. Release.

[35] Zhang Y., Koradia A., Kamato D., Popat A., Little P.J., Ta H.T. (2019). Treatment of atherosclerotic plaque: Perspectives on theranostics. J. Pharm. Pharmacol..

[36] Xie J., Lee S., Chen X. (2010). Nanoparticle-based theranostic agents. Adv. Drug Deliv. Rev..

[37] Kim Y., Lobatto M.E., Kawahara T., Lee Chung B., Mieszawska A.J., Sanchez-Gaytan B.L., Fay F., Senders M.L., Calcagno C., Becraft J. (2014). Probing nanoparticle translocation across the permeable endothelium in experimental atherosclerosis. Proc. Natl. Acad. Sci. USA.

[38] Weissleder R., Nahrendorf M., Pittet M.J. (2014). Imaging macrophages with nanoparticles. Nat. Mater..

[39] Atukorale P.U., Covarrubias G., Bauer L., Karathanasis E. (2017). Vascular targeting of nanoparticles for molecular imaging of diseased endothelium. Adv. Drug Deliv. Rev..

[40] Stigliano C., Ramirez M.R., Singh J.V., Aryal S., Key J., Blanco E., Decuzzi P.J.A.H.M. (2017). Methotraxate-Loaded Hybrid Nanoconstructs Target Vascular Lesions and Inhibit Atherosclerosis Progression in ApoE−/− Mice. Adv. Healthc. Mater..

[41] Hossain S.S., Zhang Y., Fu X., Brunner G., Singh J., Hughes T.J., Shah D., Decuzzi P. (2015). Magnetic resonance imaging-based computational modelling of blood flow and nanomedicine deposition in patients with peripheral arterial disease. J. R. Soc. Interface.

[42] Moore T.L., Hauser D., Gruber T., Rothen-Rutishauser B., Lattuada M., Petri-Fink A., Lyck R. (2017). Cellular Shuttles: Monocytes/Macrophages Exhibit Transendothelial Transport of Nanoparticles under Physiological Flow. ACS Appl. Mater. Interfaces.

[43] Key J., Palange A.L., Gentile F., Aryal S., Stigliano C., Di Mascolo D., De Rosa E., Cho M., Lee Y., Singh J. (2015). Soft Discoidal Polymeric Nanoconstructs Resist Macrophage Uptake and Enhance Vascular Targeting in Tumors. ACS Nano.

[44] Palomba R., Palange A.L., Rizzuti I.F., Ferreira M., Cervadoro A., Barbato M.G., Canale C., Decuzzi P. (2018). Modulating Phagocytic Cell Sequestration by Tailoring Nanoconstruct Softness. Acs Nano.

[45] Schottler S., Becker G., Winzen S., Steinbach T., Mohr K., Landfester K., Mailander V., Wurm F.R. (2016). Protein adsorption is required for stealth effect of poly(ethylene glycol)- and poly(phosphoester)-coated nanocarriers. Nat. Nanotechnol..

[46] Mima Y., Abu Lila A.S., Shimizu T., Ukawa M., Ando H., Kurata Y., Ishida T. (2017). Ganglioside inserted into PEGylated liposome attenuates anti-PEG immunity. J. Control. Release.

[47] Ranalli A., Santi M., Capriotti L., Voliani V., Porciani D., Beltram F., Signore G. (2017). Peptide-Based Stealth Nanoparticles for Targeted and pH-Triggered Delivery. Bioconjug. Chem..

[48] Santi M., Maccari G., Mereghetti P., Voliani V., Rocchiccioli S., Ucciferri N., Luin S., Signore G. (2017). Rational Design of a Transferrin-Binding Peptide Sequence Tailored to Targeted Nanoparticle Internalization. Bioconjug. Chem..

[49] Barchet T.M., Amiji M.M. (2009). Challenges and opportunities in CNS delivery of therapeutics for neurodegenerative diseases. Expert Opin. Drug Deliv..

[50] Kharlamov A.N., Tyurnina A.E., Veselova V.S., Kovtun O.P., Shur V.Y., Gabinsky J.L. (2015). Silica-gold nanoparticles for atheroprotective management of plaques: Results of the NANOM-FIM trial. Nanoscale.

[51] Majmudar M.D., Keliher E.J., Heidt T., Leuschner F., Truelove J., Sena B.F., Gorbatov R., Iwamoto Y., Dutta P., Wojtkiewicz G. (2013). Monocyte-directed RNAi targeting CCR2 improves infarct healing in atherosclerosis-prone mice. Circulation.

[52] Kao C.-W., Wu P.-T., Liao M.-Y., Chung I.-J., Yang K.-C., Tseng W.-Y., Yu J. (2018). Magnetic nanoparticles conjugated with peptides derived from monocyte chemoattractant protein-1 as a tool for targeting atherosclerosis. Pharmaceutics.

[53] Majmudar M.D., Yoo J., Keliher E.J., Truelove J.J., Iwamoto Y., Sena B., Dutta P., Borodovsky A., Fitzgerald K., Di Carli M.F. (2013). Polymeric nanoparticle PET/MR imaging allows macrophage detection in atherosclerotic plaques. Circ. Res..

[54] Kheirolomoom A., Kim C.W., Seo J.W., Kumar S., Son D.J., Gagnon M.K., Ingham E.S., Ferrara K.W., Jo H. (2015). Multifunctional Nanoparticles Facilitate Molecular Targeting and miRNA Delivery to Inhibit Atherosclerosis in ApoE(-/-) Mice. ACS Nano.

[55] Kim D., Hong J., Moon H.-H., Nam H.Y., Mok H., Jeong J.H., Kim S.W., Choi D., Kim S.H. (2013). Anti-apoptotic cardioprotective effects of SHP-1 gene silencing against ischemia–reperfusion injury: Use of deoxycholic acid-modified low molecular weight polyethyleneimine as a cardiac siRNA-carrier. J. Control. Release.

[56] Nakashiro S., Matoba T., Umezu R., Koga J., Tokutome M., Katsuki S., Nakano K., Sunagawa K., Egashira K. (2016). Pioglitazone-Incorporated Nanoparticles Prevent Plaque Destabilization and Rupture by Regulating Monocyte/Macrophage Differentiation in ApoE-/- Mice. Arterioscler. Thromb. Vasc. Biol..

[57] Leite A.C.A., Solano T.V., Tavares E.R., Maranhao R.C. (2015). Use of Combined Chemotherapy with Etoposide and Methotrexate, both Associated to Lipid Nanoemulsions for Atherosclerosis Treatment in Cholesterol-fed Rabbits. Cardiovasc. Drug Ther..

[58] Park D., Cho Y., Goh S.-H., Choi Y. (2014). Hyaluronic acid–polypyrrole nanoparticles as pH-responsive theranostics. Chem. Commun..

[59] Van der Valk F.M., Schulte D.M., Meiler S., Tang J., Zheng K.H., Van den Bossche J., Seijkens T., Laudes M., de Winther M., Lutgens E. (2016). Liposomal prednisolone promotes macrophage lipotoxicity in experimental atherosclerosis. Nanomed. Nanotechnol..

[60] Meneghini B.C., Tavares E.R., Guido M.C., Tavoni T.M., Stefani H.A., Kalil-Filho R., Maranhão R.C. (2019). Lipid core nanoparticles as vehicle for docetaxel reduces atherosclerotic lesion, inflammation, cell death and proliferation in an atherosclerosis rabbit model. Vasc. Pharmacol..

[61] Wu W., Feng X., Yuan Y., Liu Y., Li M., Bin J., Xiao Y., Liao W., Liao Y., Zhang W. (2017). Comparison of magnetic microbubbles and dual-modified microbubbles targeted to P-selectin for imaging of acute endothelial inflammation in the abdominal aorta. Mol. Imaging Biol..

[62] Winter P.M., Neubauer A.M., Caruthers S.D., Harris T.D., Robertson J.D., Williams T.A., Schmieder A.H., Hu G., Allen J.S., Lacy E.K. (2006). Endothelial ανβ3 integrin–targeted fumagillin nanoparticles inhibit angiogenesis in atherosclerosis. Arterioscler. Thromb. Vasc. Biol..

[63] Winter P.M., Caruthers S.D., Zhang H., Williams T.A., Wickline S.A., Lanza G.M. (2008). Antiangiogenic synergism of integrin-targeted fumagillin nanoparticles and atorvastatin in atherosclerosis. JACC Cardiovasc. Imaging.

[64] Mauricio M., Guerra-Ojeda S., Marchio P., Valles S., Aldasoro M., Escribano-Lopez I., Herance J., Rocha M., Vila J., Victor V. (2018). Nanoparticles in medicine: A focus on vascular oxidative stress. Oxid Med. Cell Longev..

[65] Harel-Adar T., Mordechai T.B., Amsalem Y., Feinberg M.S., Leor J., Cohen S. (2011). Modulation of cardiac macrophages by phosphatidylserine-presenting liposomes improves infarct repair. Proc. Natl. Acad. Sci. USA.

[66] Tang J., Baxter S., Menon A., Alaarg A., Sanchez-Gaytan B.L., Fay F., Zhao Y., Ouimet M., Braza M.S., Longo V.A. (2016). Immune cell screening of a nanoparticle library improves atherosclerosis therapy. Proc. Natl Acad. Sci. USA.

[67] Beldman T.J., Senders M.L., Alaarg A., Perez-Medina C., Tang J., Zhao Y., Fay F., Deichmoller J., Born B., Desclos E. (2017). Hyaluronan Nanoparticles Selectively Target Plaque-Associated Macrophages and Improve Plaque Stability in Atherosclerosis. ACS Nano.

[68] Cuadrado I., Piedras M.J., Herruzo I., Turpin Mdel C., Castejon B., Reventun P., Martin A., Saura M., Zamorano J.L., Zaragoza C. (2016). EMMPRIN-Targeted Magnetic Nanoparticles for In Vivo Visualization and Regression of Acute Myocardial Infarction. Theranostics.

[69] Kamaly N., Fredman G., Fojas J.J.R., Subramanian M., Choi W.I., Zepeda K., Vilos C., Yu M.Y., Gadde S., Wu J. (2016). Targeted Interleukin-10 Nanotherapeutics Developed with.a Microfluidic Chip Enhance Resolution of Inflammation in Advanced Atherosclerosis. ACS Nano.

[70] Lunov O., Syrovets T., Loos C., Beil J., Delacher M., Tron K., Nienhaus G.U., Musyanovych A., Mailander V., Landfester K. (2011). Differential uptake of functionalized polystyrene nanoparticles by human macrophages and a monocytic cell line. ACS Nano.

[71] Oh N., Park J.H. (2014). Endocytosis and exocytosis of nanoparticles in mammalian cells. Int. J. Nanomed..

[72] Veseli B.E., Perrotta P., De Meyer G.R.A., Roth L., Van der Donckt C., Martinet W., De Meyer G.R.Y. (2017). Animal models of atherosclerosis. Eur. J. Pharmacol..

[73] Lee Y.T., Lin H.Y., Chan Y.W., Li K.H., To O.T., Yan B.P., Liu T., Li G., Wong W.T., Keung W. (2017). Mouse models of atherosclerosis: A historical perspective and recent advances. Lipids Health Dis..

